# Epigenetic regulation of matrix metalloproteinases in inflammatory diseases: a narrative review

**DOI:** 10.1186/s13578-020-00451-x

**Published:** 2020-07-18

**Authors:** Jie He, Man Qin, Yingyi Chen, Ziqi Hu, Fei Xie, Ling Ye, Tianqian Hui

**Affiliations:** 1 Department of Pediatric Dentistry, Peking University School and Hospital of Stomatology, No. 22, Zhongguancun South Avenue, Haidian District, Beijing, China; 2 State Key Laboratory of Oral Diseases, West China Hospital of Stomatology, Sichuan University, Chengdu, Sichuan China

**Keywords:** Epigenetics, Matrix metalloproteinases, Inflammatory diseases, DNA methylation, Histone modification, microRNAs

## Abstract

With the acceleration of urbanization and aging and the change of lifestyle, inflammatory diseases have become one of the important threats to the health of the global population. Matrix metalloproteinases (MMPs) and tissue inhibitors of metalloproteinases (TIMPs) are involved in the metabolism of extracellular matrix (ECM). They play a key role in inflammation-related diseases. Factors such as inflammation, oxidative stress and growth factors stimulate the production of MMPs with subsequent ECM remodeling. Recently, the studies of epigenetic regulation, including the ability to predict disease progression, important pathophysiological deficiencies as well as treatment methods have been extensively discussed. This article reviews the current studies on epigenetic alterations in MMPs during inflammatory response. It is likely to provide new insights into development of efficient medications of epigenetic therapy for inflammatory diseases.

## Introduction

Inflammation is a hallmark of many chronic diseases, including rheumatoid arthritis, osteoarthritis and periodontitis [1, 2]. Although these diseases may have different etiologies, they have the similar pathological abnormalities in the immune or inflammatory responses [3, 4]. Inflammation can damage tissues and even cause necrosis, making early diagnosis and treatment of it critical [5]. So far, it has been recognized that there are three interdependent pathways involved in inflammatory soft tissue decomposition: (1) the matrix metalloproteinase (MMP)-dependent pathway; (2) the plasmid-dependent lysis reactions; (3) the phagocytic pathway (the larger matrix fragments may be disposed of by lysosomal protease lysis) [6]. Matrix metalloproteinases (MMPs) are a family of at least 28 structurally related zinc-dependent proteases. They have been classified into several subgroups: collagenases (MMP-1, -8,-13, and -18), gelatinases (MMP-2 and -9), stromelysins (MMP-3, -10, and-11), matrilysins (MMP-7 and -26), membrane-type MMPs (MMP-14, -15, -16, -17, -24 and -26), and others (MMP-12, -19, -20, -21, -22, -23,-27 and -28) [7, 8]. Their endopeptidase activity and ECM degrading properties contribute to the inflammatory pathogenesis. Four mechanisms can modulate the functional activity of MMP: (1) positive and negative transcriptional regulation of MMP gene; (2) precursor activation; (3) substrate specificity difference; (4) regulation of inhibitors of metalloproteinases [6]. In protease networks, the function of MMPs is critical for many physiological and pathological processes, including immunity, inflammation, bone resorption and wound healing [9]. Although significant effort has been invested in attempts to elucidate the identity of transcriptional factors that control the promoter activity of the MMP genes, the transcription mechanism regulating MMP, such as epigenetic regulation, is needed to be better understood during disease progression. Then, it might present more effective treatment methods [10].

In 1942, Dr Waddington proposed the term ‘epigenetics’ [11]. After the term was first used, the definition of epigenetics has come through some modifications since more molecular mechanisms regulating gene expression have been discovered. Classical genetics refers to the change of gene function caused by the change of gene sequence, such as gene mutation, which results in the hereditary change of phenotype, while epigenetics refers to the hereditary change of gene function without the change of gene DNA sequence, and eventually lead to phenotypic changes [12]. Since the initial observation that DNA methylation has undergone significant changes in most cancer cells, leading to changes in gene expression involved in various cellular functions, the importance of epigenetic mechanisms in controlling specific gene expression has been well demonstrated [13]. Epigenetics regulates gene expression mainly through DNA methylation, histone modification, ubiquitination modification, non-coding RNA regulation [14, 15]. Epigenetics have been introduced to play important roles in inflammatory diseases such as rheumatoid arthritis, pulpitis, and pulmonary tuberculosis [16–18]. This review highlights the disordered epigenetic regulatory mechanisms in regulating MMPs in inflammation-related diseases (Table 1).

**Table 1:** Epigenetic regulation on different MMP genes

Epigenetic regulation		MMPs	Inflammatory diseases	References
Histone modification	Histone methylation	MMP-1, MMP-3, MMP-9, MMP-13	Rheumatoid arthritis	[28]
		MMP-9,	Diabetes	[29]
	Histone acetylation	MMP-1 and MMP-3	Pulmonary tuberculosis	[17]
		MMP-1 and MMP-13	Arthritis	[42]
		MMP-9	Experimental autoimmune neuritis.	[38]
	Histone phosphorylation	MMP‐2 and the MT1‐MMP/TIMP‐2 complex	*Treponema denticola*	[45]
DNA methylation		MMP-9	Diabetes	[49, 50]
		MMP-2 and MMP-9	Periodontitis	[53]
		MMP-3, MMP-9, MMP-13 , MMP-14	Osteoarthritis	[56]
		MMP-14	Rheumatoid arthritis	[61]
Non-coding RNA	MicroRNAS	MMP-1 and MMP-3	Rheumatoid arthritis	[68, 70, 71]
		MMP-12	Asthma	[81]
		MMP-13	Osteoarthritis	[72]
		MMP-14	Atherosclerotic plaque	[80]
	Long non-coding RNA	MMP-2, MMP-3, MMP-9, and MMP-13	Osteoarthritis	[83]
		MMP-1, MMP-3 and MMP-9	Temporomandibular joint osteoarthritis	[84]

MMP, matrix metalloproteinase

## Histone modifications of MMPs in inflammatory diseases

Histones refer to the basic DNA-binding proteins in the nucleus of eukaryotic cells,which are the basic structural proteins of eukaryotic chromosomes [19]. It is well noted that histones play a great role in maintaining DNA structure, protecting genetic information and regulating gene expression [19]. A diverse array of histone post-translational modifications (PTMs) often occur on their flexible N- or C-terminal tails as well as globular domains, affecting interactions of histones with DNA and effector proteins [20, 21]. A bulk of literature has documented the PTMs of histone, including acetylation, phosphorylation, methylation, ubiquitination and ADP-ribosylation 3 [20, 21]. The N-terminal of core histones (H2A, H2B, H3 and H4) contains a flexible and alkaline tail region, which is the most common target for modification. At least 130 sites of modification within the N terminal tails have been detected at present. New technologies will discover more new target residues and modifications [22]. It is worth mentioning that the removal of the N-terminal tail of histones also influences structure and dynamics of chromatin that could promote or inhibit transcription activity [24]. Generally, Common active histone modifications include H3K27ac, H3K4me1/3, H3K36me3, H3K79me3, which mediate gene transcriptional activation. Common inhibitory histone modifications include H3K9me3, H3K27me 3 and H4K20me3, which mediate gene transcriptional inhibition [22]. Apart from histone methylation, acetylation and phosphorylation, the relationship between other histone modifications and MMP has not been reported. In this review, we focus on describing histone methylation, acetylation and phosphorylation of MMPs.

### Histone methylation of MMPs in inflammatory diseases

Histone methylation can produce tens of millions of modification types, which greatly increases the complexity of histone modification regulating gene expression and provides greater potential for functional regulation of histone methylation. S-adenosylmethionine is used as a donor to transfer methyl to the epsilon-amino side chains of lysine and arginine. Lysine can be modified by monomethylation, dimethylation and trimethylation, while arginine can be modified by monomethylation and dimethylation [23]. Both histone methyltransferases (HMTs) and histone demethylases (HDMs) contribute to the establishment and maintenance of different histone methylation status. The ‘effectors’ which can recognize histone methylated sites build a relationship between these modifications and their downstream processes [24, 25]. Rheumatoid arthritis (RA) is a chronic, inflammatory synovitis-dominated systemic disease with unknown etiology,which eventually leads to cartilage and bone destruction without effective treatments [26]. Some studies indicated that synovial fibroblasts (SFs) play an important role in the pathological process of RA [27]. Silencing of WD (tryptophan-aspartate) repeat domain 5 (WDR5) gene expression, which is a core subunit of complex proteins associated with SET1 (COMPASS) or COMPASS-like complexes, suppressed the levels of H3K4me3 and consequently reduced MMP-1, MMP-3, MMP-9, and MMP-13 gene expression levels. These histone methylation profiles are associated with spontaneous MMP genes in RASFs and involved in the RASF phenotypes [28].

A close crosstalk between histone methylation, histone acetylation and DNA methylation plays a critical role in the maintenance of cellular epigenetic integrity of MMP-9 in diabetes [29, 30]. Type 2 diabetes are closely related to chronic ‘inflammation’ characterized by abnormal cytokine production, increased reactants and other mediators in acute phase, and activation of inflammatory signaling pathways networks [31]. Increased H3K27me3 (histone H3 trimethylated at lysine 27) levels and more recruitment of Enhancer of zeste homolog 2 (EZH2) at the MMP-9 promoter has been detected in Hyperglycemia. And the enzyme activity of EZH2 has been enhanced. Inhibition of EZH2 attenuated recruitment of both DNA methylating (DNMT1) and hydroxy methylating (TET2) enzymes and 5 hydroxymethyl cytosine at the same region of the MMP-9 promoter, preventing increase in MMP-9 transcription and mitochondrial damage [29].

### Histone acetylation of MMPs in inflammatory diseases

A large amount of studies have proved that histone acetylation is related with gene activation [32, 33]. When histone acetyltransferase (HAT) transfers the acetyl group of acetyl-coenzyme A(CoA))to the ε-amino group of specific lysine residues at the amino end of histone, the negatively charged acetyl group breaks the original potential balance of histone, resulting in the relaxation and unfolding of the structure of peripheral DNA sequence. This promotes the contact between transcription factors and DNA sequences, at which time the transcription of DNA sequences is enhanced [22]. Histone acetylation is characterized by specific activation of specific gene transcription processes because HAT is divided into 15 types and each type of HAT tends to target specific lysine sites on one or more histones [34]. Histone deacetylase (HDAC) has the function of removing acetyl group. When HDAC removes acetyl group from lysine residue of histone, histone restores its positive charge, increases the attraction between histone and DNA, and makes transcription factors difficult to approach promoter, thus reversely inhibiting the transcription of specific genes [35]. The HDACs are grouped into four classes: class I (HDACs 1,2,3, and 8), class II (HDACs 4,5,6,7,9, and10), class III (SIRT1, SIRT2, SIRT3, SIRT4, SIRT5, SIRT6, and SIRT7), and class IV (HDAC11) [36, 37]. Histone acetyltransferase (HAT), histone deacetylase inhibitor (HDACI) and HDAC siRNA has been used to study the role of histone acetylation in inflammation-induced MMP expression (Table 2). Moores et al. found that non-selective inhibition of HDAC activity attenuated the induction of MMP-1/-3 by Mycobacterium tuberculosis (Mtb) stimulated macrophages and normal human bronchial epithelial cells(NHBEs) [17]. Inhibition of histone acetyltransferase (HAT) activity also effectively repressed MMP-1/-3 secretion by Mtb-infected macrophages [17]. MS-275 (Entinostat), another new HDACi, has been shown to significantly reduce the expression of MMP-9 in a preclinical model of experimental autoimmune encephalomyelitis (EAE) [38]. What’s more, HDACs can inhibit the expression of MMPs induced by inflammatory cytokines and other mediators [39–42]. After the treatment with interleukin (IL)-1β, histone H4 acetylation increased at AP‐1–specific promoter sites of MMP-1 and MMP-13 [9]. Both MMP-1 and MMP-13 proteins were potently induced by treatment with IL-1α and oncostatin M(OSM) and this induction was repressed by both TSA (Trichostatin A) and NaBy (sodium butyrate, HADCi) [42]. In addition, TSA attenuated the induction of MMP-1 and -13 by FGF2 and IL-1β in a dose dependent manner [41]. In contrast, overexpression of HDAC4 partially blocks the effect of IL-1β on MMP-1, MMP-3, MMP-13 [43]. In diabetes, lysine demethylase (LSD1) enhanced glucose-induced decrease in H3K9me2 and increase in p65 at the MMP-9 promoter, and this demethylation releases lysine 9 for acetylation. Acetylation of H3K9 opens up the chromatin and increases the accessibility of recruit transcription factors and activates the transcription of MMP-9 [30]. Furthermore, the inhibition of Sirtuin 1 (Sirt1), which is a deacetylase and play a central role in the acetylation-deacetylation of p65, increasing MMP-9 transcription [44].

**Table 2:** Role of HDAC inhibitors and HAT inhibitors in MMPs

	Target genes	Inflammatory diseases	References
HDAC inhibitors			
MS-275	MMP-1↓	Pulmonary tuberculosis	[17]
	MMP-9↓	Experimental autoimmune neuritis.	[38]
CBHA	MMP-1↓	Pulmonary tuberculosis	[17]
TSA	MMP-1/-3↓		[17]
TSA and NaBy	MMP-1 and MMP-13↓	Arthritis	[42]
HAT inhibitors			
AA	MMP-1/-3↓	Pulmonary tuberculosis	[17]
HATi II			

HDAC, histone deacetylases; HAT, histone acetylation; CBHA, m-Carboxycinnamic Acid bis-Hydroxamide; TSA, Trichostatin A; NaBy, sodium butyrate; AA, Anacardic acid; MMP, matrix metalloproteinase

### Histone phosphorylation of MMPs in inflammatory diseases

Histone phosphorylation refers to the process of transferring the phosphoryl group of ATP to histone-specific amino acids through protein phosphokinase. Histone phosphorylation occurs mostly on serine(S), threonine(T) and tyrosine(Y) residues and constitutes an critical part of the “histone code,” or combinatory function of PTMs on chromatin Pretreatment or post‐treatment of PDL cells with PF‐03814735 (histone phosphorylation inhibitor) significantly prevented or reversed the increase of MMP‐2 and the MT1‐MMP/TIMP‐2 complex mediated by *Treponema denticola* (*T. denticola*) [45].

Different types of histone modifications correspond to different enzymes, including the establishment of modified enzymes, such as methyltransferase, acetyltransferase, ubiquitinase, etc., and the removal of modified enzymes, such as demethylase, deacetylase, etc. These regulatory molecules can recognize different histone modifications, then regulate transcription, change downstream molecular events, and ultimately affect the progress of inflammatory diseases.

## DNA methylation of MMPs in inflammatory diseases

DNA methylation is a biochemical reaction in which methyl groups are added to cytosine or adenine DNA nucleotides by DNA methyltransferases (DNMTs), and it is one of the stable epigenetic markers [46]. DNA methylation which happens on the fifth position of cytosine (5-methylcytosine, 5mC) is mostly located at the position of Cytosine-phosphate-guanine (CpG) dinucleotides. The pattern of DNA methylation is mainly divided into de novo methylation and the maintenance methylation. The former is catalyzed by DNA methyltransferases (DNMT) DNMT3A and DNMT3B, while the maintenance methylation is catalyzed by DNMT1 [47, 48].

DNA methylation is the most widely studied epigenetic mechanism in diabetes. In diabetes, the expression of MMP-9 in retina and capillaries is increased, which promotes the development of the disease. It was found that glucose could increase the binding of DNMT1 and TETs2 at MMP-9 promoter region. At the same time, the level of 5hmC in this region increased, while the level of 5mC decreased. Therefore, the transcription of MMP-9 in diabetic retinopathy is maintained to some extent through the process of DNA methylation-hydroxymethylation [49]. Growth arrest and DNA damage-inducible 45a (GADDD45a) is a DNA demethylation regulatory protein, which up-regulates significantly in skin tissues of patients with diabetic foot ulcer and in human keratinocyte (HaCaT) cells exposed to advanced glycation end products [50]. Liyan Zhou et al. showed that Gadd45a mediated demethylation of diabetic HaCaT cells and diabetic rat skin by binding to the promoter of MMP-9. Their results revealed that GADD45A is essential for MMP-9 promoter demethylation and wound healing in diabetes [50].

Previous studies have shown that DNA methylation plays a key role in the pathogenesis of periodontitis [51]. High-throughput DNA analysis suggests that there is a significant change in DNA methylation between healthy and periodontitis cases among the genes associated with immune inflammation [51]. *T. denticola*, a well-known periodontal pathogen, can decrease the methylation level of MMP2 promoter and activate pro-MMP-2 (prozymogen form of MMP-2) in periodontal ligament cells [52]. This indicated that *T. denticola* promotes the loss of supporting tissues in periodontitis through epigenetic mechanisms. In addition, the methylation level of MMP-9 CpG islands was positively correlated with the severity of chronic periodontitis [53]. High MMP-9 mRNA expression and low DNA methylated profiles of the MMP-9 gene have been found in periapical inflammatory lesions [54]. In addition, the methylation level of MMP-9 CpG islands was positively correlated with the severity of chronic periodontitis [53].

Osteoarthritis (OA) refers to a heterogeneous disease with a wide range of underlying pathologies that ultimately lead to joint damage [55]. It has been found that the CpG site located near the transcriptional initiation point of MMP-9 promoter in hip dysplasia (DDH) is severely demethylated compared with the control group [56]. A loss of DNA methylation of the promoters of various OA associated genes have been founded in OA chondrocytes, including matrix metalloproteinase 3 (MMP-3), MMP-9, MMP-13, ADAMTS-4 (a disintegrin and metalloproteinase with thrombospondin motifs), and interleukin-1 (IL-1) [57–60]. Emmanuel karouzakis et al. reported low levels of global DNA methylation in RA synovial tissue and in cultured RASFs [61]. The 5-azacytidine (5-azaC), an inhibitor of DNMTs, upregulates the expression of MMP-14 o in both OASFs and RASFs [61].

So far, DNA methylation has been extensively studied in epigenetic events. DNA methylation is chemically stable, easy to amplify, easy to detect, and is an indispensable biomarker. A large number of studies have shown that the progress of inflammatory diseases is closely related to changes in DNA methylation patterns and abnormal DNA methylation. It is worth noting that the balance between DNA hypermethylation and demethylation needs to be maintained, and how to effectively and appropriately regulate gene expression by adjusting the balance between the two deserves great attention.

## Non-coding RNAS mediated MMPs in inflammatory diseases

The term non-coding RNA (ncRNA) is commonly employed for RNA that does not encode a protein, but this does not mean that such RNAs do not contain information nor have function [62]. The ncRNAs could be classified based on size: small (around 20 base pairs (bp)), intermediate (less than 200 bp) and long (longer than 200 bp) [63]. This review is mainly focused on the microRNAs (miRNAs) and long non-coding RNA (lncRNA).

### MicroRNAs mediated MMPs in inflammatory diseases

Small non-coding RNAs, known as miRNAs, are essential regulators of gene expression in animal genomes [64]. Generally, they have 21–25 nucleotide lengths and are powerful post transcriptional regulators because they inhibit gene expression by hybridizing with the 3′-untranslated region (3′-UTR) of target messenger RNAs [65, 66]. MicroRNAs not only play a key role in a wide range of biological and cellular processes, such as development, cell proliferation, and apoptosis, but also participate in many pathological processes [67] (Table 3). Microarray analysis showed that both miR-155 and miR-146a are highly expressed in RASFs [68]. The overexpression of microRNA-155 inhibits the expression of MMP-3 and suppress the induction of Toll-like receptor (TLR) ligands and cytokines to MMP-1 and MMP-3 in RASF. MicroRNA-155 acts as a protective microRNA, which locally reduces the expression of MMP, thus preventing excessive tissue damage caused by inflammation [68]. In the same group, 260 miRNAs were screened to detect differentially expressed miRNAs in RASFs [69]. As a result, it founded that microRNA-203 was highly expressed in RASF. The overexpression of miR-203 significantly upgrades the secretion of MMP-1. Another research recovered that overexpression of miR-19b decreased the expression of MMP-3, and has been recognized as a negative regulator of inflammation in RA [70]. Other miRNAs upregulated in RASF and implicated with the production of MMP-1 is miR-18a [71]. In addition, miR-146a, as a negative regulator in the inflammatory response, inhibits MMP-13 in human articular chondrocytes and other cell types [72–74]. The repression of MMP-13 in OA patients may also be mediated by other miRNAs, including miR-22, miR-320, miR-127-5p and miR-27a/b [75–79].

**Table 3:** microRNAs mediated MMPs in inflammatory diseases

MicroRNAs	Target genes	Inflammatory diseases	References
miR-155	MMP-1 and MMP-3↓	Rheumatoid arthritis	[68]
miR-203/miR-18a	MMP-1↑	Rheumatoid arthritis	[69, 71]
miR-19b	MMP-3↓	Rheumatoid arthritis	[70]
miR-146a/miR-320/miR-27/miR‐127‐5p/miR-22	MMP-13↓	Osteoarthritis	[75–79]
miR-24	MMP-14↓	Atherosclerotic plaque	[80]
miR-672/miR-143	MMP-12↓	Asthma	[81]

miR, microRNA; MMP, matrix metalloproteinase

MicroRNA-24 expression in these atherosclerotic plaques was inversely related to MMP-14 protein expression [80]. MMP-12 is highly correlated with inflammatory diseases such as asthma. Bioinformatics analysis suggested that MMP-12 mRNA had potential binding sites for mir-672 and mir-143, which might be candidate target genes for them. Matrix metalloproteinase-12 (MMP-12) is considered to be highly associated with inflammatory diseases [81].

### Long non-coding RNAs mediated MMPs in inflammatory diseases

Long non-coding RNA (lncRNA) is a non-coding RNA with a length of more than 200 nucleotides. It also engage in numerous biological processes across every branch of life [82].

Compared with normal chondrocytes, higher expression of lncRNA growth arrest-specific 5 (GAS5) is found in chondrocytes from OA patients. Additionally, overexpression of GAS5 induces the expression of MMP-2, MMP-3, MMP-9, and MMP-13 [83]. Knockdown of HOTAIR (lncRNAs HOX antisense intergenic RNA) in Temporomandibular Joint(TMJ) OA could reverse the IL-1β-stimulated expressions of MMP-1, MMP-3 and MMP-9. These data provide new insight into the mechanisms of chondrocytes destruction in TMJ OA [84].

NcRNAs are involved in various stages of the occurrence and development of inflammatory diseases. Through different mechanisms of action, they lead to changes in the expression and activity of matrix metalloproteinases, thus affecting the outcome of inflammatory diseases.

## Conclusion

Inflammatory diseases include many infectious diseases and autoimmune diseases, cardiovascular diseases, diabetes and other chronic non-communicable major diseases. These diseases are spreading globally and have evolved into important public health problems that seriously endanger human health and social and economic sustainable development. To study the common mechanism of inflammation will provide new strategies for the prevention, diagnosis and treatment of diseases, which has attracted more and more attention from governments and societies.

This review demonstrated the major epigenetic mechanisms, including DNA methylation, histone modifications, and non-coding RNAs, could regulate gene expression of MMPs and affect progression of inflammatory diseases. At present, many breakthroughs have been made in the study of epigenetics of MMPs, but whether epigenetics can really be used as a target for disease diagnosis, prognosis and treatment remains to be explored in depth.

## Data Availability

Not applicable.

## Abbreviations

AA: Anacardic acid
5-azaC: 5-azacytidine
CBHA: m-Carboxycinnamic Acid bis-Hydroxamide
CpG: Cytosine-phosphate-guanine
DNMT: DNA methylating
DNMTs: DNA methyltransferases
EAE: Experimental autoimmune encephalomyelitis
ECM: Extracellular matrix
EZH2: Enhancer of zeste homolog 2
GADDD45a: Growth arrest and DNA damage-inducible 45a
GAS5: Growth arrest-specific 5
HaCaT: Human keratinocyte
HAT: Histone acetyltransferase
HASMCs: Human aortic smooth muscle cell
HDAC: Histone deacetylase
HDMs: Histone demethylases
H3K27me1: H3 mono methylation at lysine 27
H3K27me3: Histone H3 trimethylated at lysine 27
HMTs: Histone methyltransferases
IL-1: Interleukin-1
LSD1: Lysine demethylase
MMPs: Matrix metalloproteinases
MiR: MicroRNA
Mtb: Mycobacterium tuberculosis
NcRNA: Non-coding RNA
NHBEs: Normal human bronchial epithelial cells
OA: Osteoarthritis
OSM: Oncostatin M
oxLDL: Oxidized low-density lipoprotein
pro-MMP-2: Prozymogen form of MMP2
PTMs: Post-translational modifications
RA: Rheumatoid arthritis
SFs: Synovial fibroblasts
Sirt1: Sirtuin 1
*T. denticola*: *Treponema denticola*
TMJ: Temporomandibular Joint
TIMPs: Tissue inhibitors of metalloproteinase
TLR: Toll-like receptor
TSA: Trichostatin A
3′-UTR: 3′-untranslated region
WDR5: WD (tryptophan-aspartate) repeat domain 5
